# Effects of Muscle Fatigue and Recovery on Complexity of Surface Electromyography of Biceps Brachii

**DOI:** 10.3390/e23081036

**Published:** 2021-08-12

**Authors:** Fuyuan Liao, Xueyan Zhang, Chunmei Cao, Isabella Yu-Ju Hung, Yanni Chen, Yih-Kuen Jan

**Affiliations:** 1 Department of Biomedical Engineering, Xi’an Technological University, Xi’an 710021, China; liaofuyuan1024@163.com; 2 Rehabilitation Engineering Lab, Department of Kinesiology and Community Health, University of Illinois at Urbana-Champaign, Champaign, IL 61820, USA; mrkx88@126.com; 3 Division of Sports Science and Physical Education, Tsinghua University, Beijing 100084, China; caocm@tsinghua.edu.cn; 4 Department of Nursing, Chung Hwa University of Medical Technology, Tainan 717302, Taiwan; isabella@ms.hwai.edu.tw; 5 Xi’an Children’s Hospital and The Affiliated Children’s Hospital of Xi’an Jiaotong University, Xi’an 710021, China; chenyannichil@163.com

**Keywords:** muscle fatigue, muscle recovery, regularity degree, surface electromyography, sample entropy

## Abstract

This study aimed to investigate the degree of regularity of surface electromyography (sEMG) signals during muscle fatigue during dynamic contractions and muscle recovery after cupping therapy. To the best of our knowledge, this is the first study assessing both muscle fatigue and muscle recovery using a nonlinear method. Twelve healthy participants were recruited to perform biceps curls at 75% of the 10 repetitions maximum under four conditions: immediately and 24 h after cupping therapy (−300 mmHg pressure), as well as after sham control (no negative pressure). Cupping therapy or sham control was assigned to each participant according to a pre-determined counter-balanced order and applied to the participant’s biceps brachii for 5 min. The degree of regularity of the sEMG signal during the first, second, and last 10 repetitions (Reps) of biceps curls was quantified using a modified sample entropy (*E_ms_*) algorithm. When exercise was performed immediately or 24 h after sham control, *E_ms_* of the sEMG signal showed a significant decrease from the first to second 10 Reps; when exercise was performed immediately after cupping therapy, *E_ms_* also showed a significant decrease from the first to second 10 Reps but its relative change was significantly smaller compared to the condition of exercise immediately after sham control. When exercise was performed 24 h after cupping therapy, *E_ms_* did not show a significant decrease, while its relative change was significantly smaller compared to the condition of exercise 24 h after sham control. These results indicated that the degree of regularity of sEMG signals quantified by *E_ms_* is capable of assessing muscle fatigue and the effect of cupping therapy. Moreover, this measure seems to be more sensitive to muscle fatigue and could yield more consistent results compared to the traditional linear measures.

## 1. Introduction

Muscle fatigue is described as the exercise-induced reduction in capacity to generate force or power output [1]. It is caused not only from peripheral changes in muscles, but also from an inadequate neural drive to the muscles [2]. At the central level, reduced cell excitability within the cerebral motor cortex leads to decreases in the number of recruited motor units and their discharge rate. At the peripheral level, metabolic and structural changes in muscles result in an altered neuromuscular transmission of muscle action potentials, as well as decreased contractile strength of the muscle fibers [3]. Various interventions, such as cupping therapy and heating interventions, are used to improve muscle recovery from fatigue [4]. However, many studies have been conducted based on subjective outcomes, such as perceived fatigue and soreness, and there is no consensus in the literature regarding effective management of muscle fatigue [4].

Various methods have been proposed to assess muscle fatigue by measuring biochemical or physiological changes in fatigued muscles. Surface electromyography (sEMG) can be used to noninvasively monitor muscle fatigue in a real-time manner [5]. Its applicability is based on the fact that myoelectric alterations can be revealed by the sEMG signal [6,7]. To date, indices that have been proposed to characterize sEMG signals for assessing muscle fatigue can be grouped into two classes: linear and nonlinear indices [8]. The most commonly used linear indices include root mean square (RMS), mean frequency (MNF), median frequency (MDF), and spectral moment ratio (SMR) [5]. It has been well established that a decrease in MNF or MDF and an increase in SMR indicate a shift of sEMG from high to low frequencies, which is associated with decreased conduction velocity of action potentials in fatigued muscles [9,10], Moreover, spectral indices (e.g., MNF and MDF) exhibit more consistent variations compared to amplitude-based indices (e.g., RMS).

The use of linear indices of sEMG for assessing muscle fatigue is based on the assumption that sEMG can be conceived as a Gaussian random process [9]. However, there is evidence that sEMG is nonlinear in nature and expresses deterministic chaos [11,12]. The complex sEMG patterns change with muscle activation conditions [13]. In this context, nonlinear time-series analyses were introduced to assess complexity features of sEMG. A common finding of the reported studies was that muscle fatigue results in a loss of complexity of the sEMG signal [9]. However, most of the complexity measures require very large datasets to attain reliable results [9]. To address this problem, a number of studies employed sample entropy ($E_{s}$) and fuzzy approximate entropy ($E_{f}$) to quantify the complexity of sEMG signals [14,15,16,17], because these two indices have been demonstrated to be largely independent of the data length [14,18]. However, these studies focused on analyzing sEMG signals during isometric muscle contractions [14,15,16]. In particular, it was reported that $E_{f}$ showed a decreasing trend similar to that of MNF with the development of muscle fatigue [14]. In summary, although some nonlinear methods seem to be efficient in detecting alterations of sEMG signals related to muscle fatigue, it is unclear how changes in nonlinear features of sEMG signals differ from changes in linear features with muscle fatigue during dynamic contractions, as well as during muscle recovery.

In this study, we employed a nonlinear method, modified sample entropy ($E_{ms}$) algorithm [19], to quantify the degree of regularity of sEMG signals for assessing muscle fatigue and recovery from fatigue after cupping therapy. To the best of our knowledge, this is the first study assessing both muscle fatigue and recovery using a nonlinear method. We hypothesized that $E_{ms}$ would be capable of revealing alterations of sEMG with the development of muscle fatigue and muscle recovery after cupping therapy and would be more sensitive to changes in sEMG compared to the traditional linear indices.

## 2. Methods

This study was approved by the institutional review board of The University of Illinois at Urbana-Champaign (#20423). This was a study within a larger research project on assessing the effectiveness of cupping therapy on improving blood flow [20,21]. The complexity analysis of the sEMG signal has not been reported elsewhere.

### 2.1. Participants

Twelve healthy adults (6 males and 6 females) were recruited. Their demographic data were (mean ± standard deviation): age 27.5 ± 6.3 years and body mass index 22.3 ± 2.6 kg/m^2^. The exclusion criteria included diagnosed ischemic heart diseases, hypertension (SBP ≥ 140 mmHg or DBP ≥ 90 mmHg), vascular disease, diabetes mellitus, or neuromuscular disorders. Participants who experienced adverse reactions to exercise or cupping therapy were also excluded. Informed written consent was obtained from each participant prior to any tests.

### 2.2. Study Design

**Figure 1:**
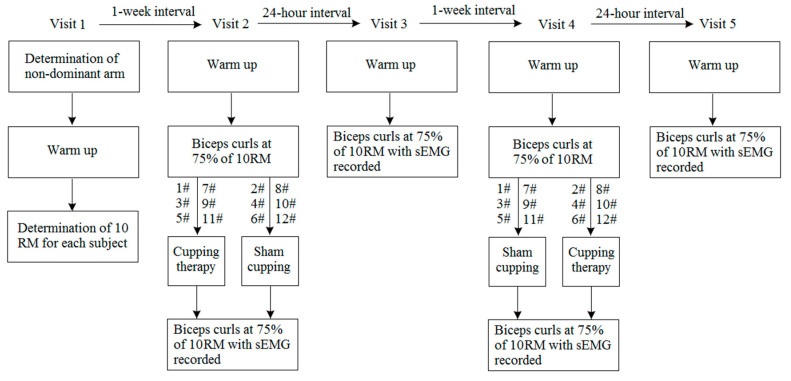
Study design and experimental procedure of this study. 10 RM, ten-repetition maximum.

A repeated-measures design was adopted in this study, consisting of five successive visits (Figure 1). During the second visit, half of the subjects received cupping therapy, and the other half received sham control according to a pre-determined order; during the fourth visit, each subject received another intervention. The counter-balanced order was aimed to reduce the carry-over effect of the interventions. This design allowed researchers to examine myoelectric manifestations of muscle fatigue during biceps curls in each subject under four conditions: immediately, and 24 h after cupping therapy, as well as after sham control. Cupping therapy was implemented by applying a negative pressure of 300 mmHg to the biceps brachii for 5 min using a cup with an inner diameter of 45 mm and a rim width of 4 mm, while sham control involved placing the cup without pressure on the same site for 5 min [22,23]. The choices of the amount of negative pressure, cup size, and duration of the interventions were based on previous studies [22,23]. To minimize the influence of physical activities in daily life, all subjects performed biceps curls using their non-dominant arm in a sitting position on a bench.

### 2.3. Experimental Procedure

The first visit was aimed to determine the ten-repetition maximum (10 RM). After a 5-min warm-up (biceps curls without load) followed by a 3-min rest, the subject performed biceps curls using dumbbells with different weights during several trials [24]. In the first trial, the initial weight was adjusted according to the subject’s estimation. If the subject could complete more (or less) than 10 Reps, the weight would be increase (or decrease) in the next trial. This process was repeated until the subject could complete exactly 10 Reps.

During the second and fourth visits, after a 5-min warm up followed by a 3-min rest, the subject performed biceps curls at 75% of 10 RM at a tempo of 15 Reps per minute [25,26]. Each 10 Reps was separated by a 30-s rest. This process was repeated until task failure. After this fatigue protocol, the subject received cupping therapy or sham control according to the pre-determined order. Then, the subject completed the same fatigue protocol, during which the sEMG signal from the biceps brachii was recorded at a sampling rate of 1000 Hz using a bipolar electrode configuration (circular Ag-AgCl wet gel electrodes, 11-mm diameter, 11-mm inter-electrode distance) (EL507, Biopac System Inc., Goleta, CA, USA). The electrodes were placed on the line between the media acromion and the cubital fossa with the midpoint of the center to center-line at 1/3 of the way from the cubital fossa [27]. Prior to the electrode placement, the skin was shaved and cleaned with alcohol. After data collection, the raw sEMG signal was processed using an adaptive algorithm [28] to remove the power frequency interference without compromising the actual signal within the interference frequency bands.

The third and fifth visits were aimed to examine the delayed effect of cupping therapy on reducing muscle fatigue. After a 5-min warm up followed by a 3-min rest, the subject performed the same fatigue protocol with the sEMG signal from the biceps brachii being collected.

### 2.4. Data Analysis

#### 2.4.1. Modified Sample Entropy

Sample entropy ($E_{s}$) is a commonly used measure of the regularity (irregularity) degree of a time series, defined as the negative natural logarithm of the conditional probability that two m-point sequences within a tolerance r remain within the tolerance at the next point [18]. A smaller (lager) value of $E_{s}$ indicates a higher degree of regularity (irregularity). An outstanding advantage of $E_{s}$ is its insensitivity to the data length and therefore suitable for analyzing short datasets [18]. However, for temporally correlated data, $E_{s}$ is dependent on the sampling rate [19]. Due to the fact that physiological time series are usually long-range correlated, the dependence of $E_{s}$ on the sampling rate can lead to different interpretations of a given physiological process in terms of “regularity” due to different sampling rates. To address this problem, we modified the $E_{s}$ algorithm by introducing a lag between the successive data points of the sequences to be compared, which could be estimated through the auto mutual information function of the time series [19]. Here, we briefly present the $E_{ms}$ algorithm below. For a time series of length N, {x(i),i=1,…,N}, its m-point sequences are defined as

(1) $$x_{m}^{\tau }(i)=\{x(i+k\tau ),0\leq k\leq m-1\},\;1\leq i\leq N-m\tau ,$$

where τ is a lag. The distance between two sequences $x_{m}^{\tau }(i)$ and $x_{m}^{\tau }(j)$ is defined as

(2) $$d[x_{m}^{\tau }(i),x_{m}^{\tau }(j)]=\max\{|x(i+k\tau )-x(j+k\tau )|,0\leq k\leq m-1\},\;1\leq i,j\leq N-m\tau ,\;|j-i|>\tau .$$

For a specific sequence $x_{m}^{\tau }(i)$, suppose the total number of sequences $x_{m}^{\tau }(j)$ satisfying |j−i|>τ is $n_{i}$ and $n_{i}^{m}(r)$ of them satisfy $d[x_{m}^{\tau }(i),x_{m}^{\tau }(j)]<r$, where r is a tolerance, then $C_{i}^{m}(r)=n_{i}^{m}(r)/n_{i}$ represents the probability that any sequence $x_{m}^{\tau }(j)$, |j−i|>τ, is within the tolerance of $x_{m}^{\tau }(i)$, and $C^{m}(r)=\sum_{i=1}^{N-m\tau }C_{i}^{m}/(N-m\tau )$ represents the probability that any two sequences $x_{m}^{\tau }(i)$ and $x_{m}^{\tau }(j)$, |j−i|>τ, are within the tolerance. Likewise, $C^{m+1}(r)$ represents the probability that any two (m+1)-point sequences $x_{m+1}^{\tau }(i)$ and $x_{m+1}^{\tau }(j)$, |j−i|>τ, are within the tolerance. In this way, $E_{ms}$ is defined as

(3) $$E_{ms}(m,r,\tau )=\lim_{N\to \infty }-\ln\frac{C^{m+1}(r)}{C^{m}(r)},$$

which is estimated by the statistic

(4) $$E_{ms}(m,r,\tau ,N)=-\ln\frac{C^{m+1}(r)}{C^{m}(r)}$$

**Figure 2:**
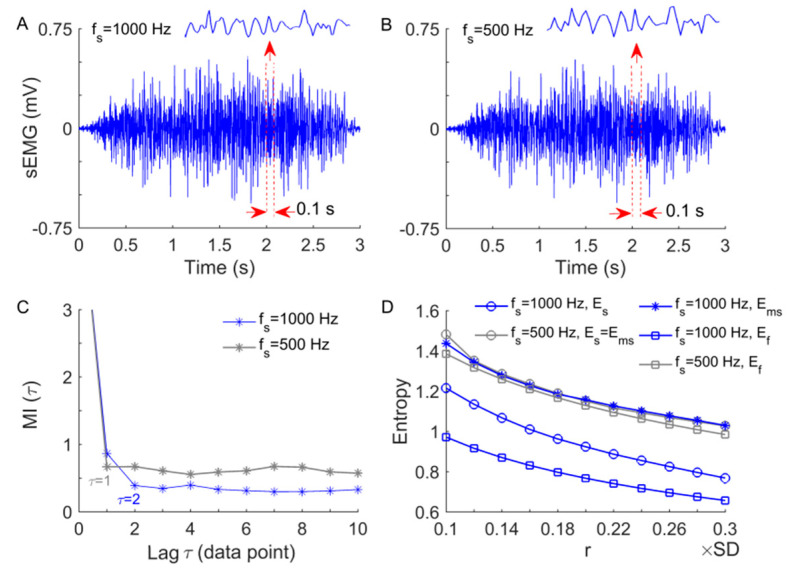
Illustration of the independence of $E_{ms}$ and dependence of $E_{s}$ and $E_{f}$ on the sampling rate ($f_{s}$ ). (**A**) A segment of a sEMG signal sampled at 1000 Hz during a repetition of biceps curls. (**B**) The downsampled signal segment by a factor of 2 ($f_{s}$ = 500 Hz). (**C**) The lags determined by the first minima of the auto mutual information (MI) functions are τ = 2 and τ = 1 when $f_{s}$ = 1000 Hz and $f_{s}$ = 500, respectively. (**D**) $E_{ms}$ yields almost identical values for the signal segments sampled at 1000 and 500 Hz but $E_{s}$ and $E_{f}$ do not, where the parameters m = 2 and r = (0.1–0.3) × SD are used. Note that when $f_{s}$ = 500 Hz, $E_{ms}$ retrieves $E_{s}$.

**Figure 3:**
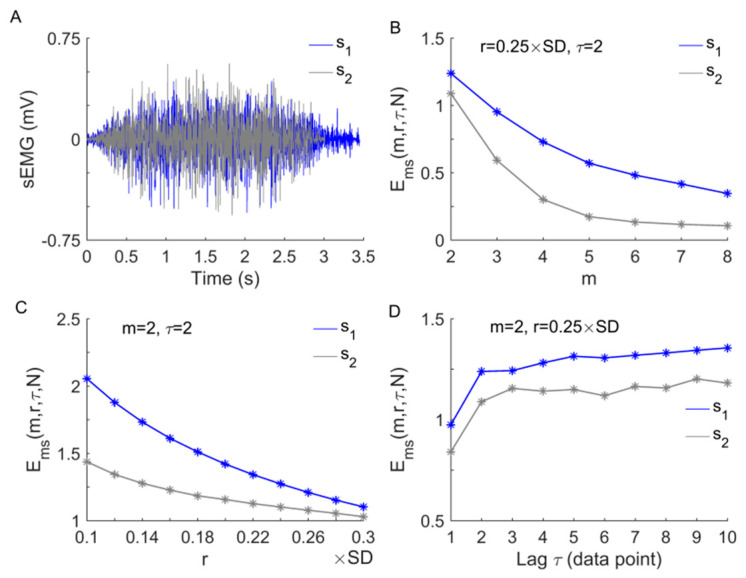
Relative consistency of $E_{ms}(m,r,\tau ,N)$ for varying values of m, r, and τ. (**A**) Two segments of a sEMG signal corresponding to a repetition of the first 10 Reps ($s_{1}$ ) and a repetition of the second 10 Reps ($s_{2}$ ), respectively. (**B**) $E_{ms}$ of $s_{1}$ is larger than that of $s_{2}$ for m = 2 to 8, where r = 0.25 × SD (standard deviation of the signal segment) and τ = 2. (**C**) $E_{ms}$ of $s_{1}$ is larger than that of $s_{2}$ for r = 0.1 × SD to 0.3 × SD, where m = 2 and τ = 2. (**D**) $E_{ms}$ of $s_{1}$ is larger than that of $s_{2}$ for multiple values of τ, where m = 2, r = 0.25 × SD. Particularly, $E_{ms}$ initially rises with τ increasing from 1 to 2 and then reaches a plateau.

The performance of $E_{ms}$ has been tested extensively in our previous studies using a simulated time series as well as skin blood-flow data [19,29,30,31]. The results showed that $E_{ms}$ is independent of the sampling rate [19], largely independent of N [31], and relative consistent for varying values of m and r [19,29,31]. When using multiple values of τ, $E_{ms}$ is actually a multiscale entropy measure [29,30]. Here, we further demonstrate that for sEMG signals, $E_{ms}$ also does not depend on the sampling rate but $E_{s}$ and $E_{f}$ do (Figure 2) and that $E_{ms}$ shows relative consistency for varying values of m and r (Figure 3B,C). When using multiple values of τ, $E_{ms}$ initially rises with τ increasing from 1 to 2, then reaches a plateau for larger values of τ (Figure 3D). This implies that it may be unnecessary to quantify the degree of regularity of the sEMG signals at multiple scales.

#### 2.4.2. Application of $E_{ms}$ to sEMG Data

**Figure 4:**
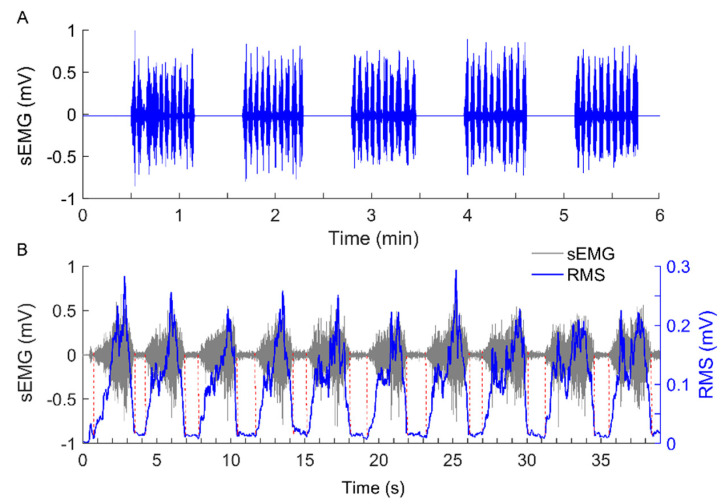
(**A**) A sEMG signal recorded from the biceps brachii of a subject who performed biceps curls at 75% of 10 RM. (**B**) Ten segments were extracted from the sEMG signal during 10 Reps of biceps curls (referred to as an epoch), each of which corresponded to a repetition. The boundary points of the segments were obtained by inspecting the plateau intervals of the root mean square (RMS) values of the signal computed using a 0.1-s (100-point) moving window with a step of 0.02 s.

To investigate whether $E_{ms}$ of the sEMG signal is capable of characterizing myoelectric alterations of muscle fatigue during dynamic contractions and, therefore, whether this measure could be used to assess the effect of cupping therapy, we applied $E_{ms}$ to the sEMG signal during the first, second, and last 10 Reps of biceps curls, thereby computing its relative change in $E_{ms}$ during the second and last 10 Reps. The method for computing $E_{ms}$ of the sEMG signal during 10 Reps of biceps curls (referred to as a signal epoch) is illustrated in Figure 4. After being filtered using a 4rd order Butterworth band pass filter (20–450 Hz) [9,32], 10 segments were extracted from the signal epoch, each of which corresponded to a repetition of exercise (Figure 4B). Given the tempo of exercise being 15 Reps per minute, all signal segments lasted less than 4 s. Such a duration is comparable to those adopted by previous studies in spectral analysis or entropy computation [8,10,14]. Then, $E_{ms}$ was computed for each segment. Their mean reflects the overall degree of regularity of the signal epoch. The relative change in $E_{ms}$ of the second (last) epoch was defined as $[(E_{ms}-E_{ms}^{\left(1\right)})/E_{ms}^{\left(1\right)}]\times 100\%$, where $E_{ms}^{\left(1\right)}$ is the entropy value of the first epoch. In the computation of $E_{ms}$ of all signal segments, the parameters m = 2, r = 0.25 × SD, and τ = 2 were used according to the previous studies [9] and our testing results.

To get an insight into the role of nonlinearity of sEMG during the development of muscle fatigue, and into the influence of cupping therapy on nonlinearity of sEMG, we performed the following experiments. For each segment of the sEMG signal during the first and second 10 Reps, we computed $E_{ms}$ for 30 phase-randomized surrogate time series. Then, the $E_{ms}$ values were averaged across 30 surrogate time series and across 10 segments. The difference in $E_{ms}$ between the sEMG signal and surrogate time series, denoted as $\Delta E_{ms}$, reflects the nonlinearity of the signal.

#### 2.4.3. Linear Analysis of sEMG Data

To compare the sensitivity of $E_{ms}$ in detecting myoelectric alterations of muscle fatigue with that of linear measures, we also computed MNF, MDF, and SMR and their relative changes for the same signal epochs from which $E_{ms}$ and its relative change were computed. In the computation of SMR, the higher order of the spectral moments was selected as 5 [8].

#### 2.4.4. Statistical Analysis

One-way ANOVA with paired *t*-tests were performed to examine the differences in $E_{ms}$, MNF, MDF, and SMR of the sEMG signal between the first, second, and last 10 Reps of biceps curls during the same visit. The differences in the relative changes in these measures between sham control and cupping therapy, as well as between the second and last 10 Reps were examined using *t*-tests. Prior to implementing the above tests, the normality of the results was checked using Shapiro–Wilk tests. All statistical analyses were performed using the SPSS (Version 26, Chicago, IL, USA).

## 3. Results

All subjects performed biceps curls with their non-dominant arms using loads, i.e., 75% of 10 RM, of 5.7 ± 1.8 kg (mean ± standard deviation). The numbers of 10 Reps for exercise immediately and 24 h after the interventions were 7.3 ± 2.9 and 4.6 ± 1.4, respectively.

**Figure 5:**
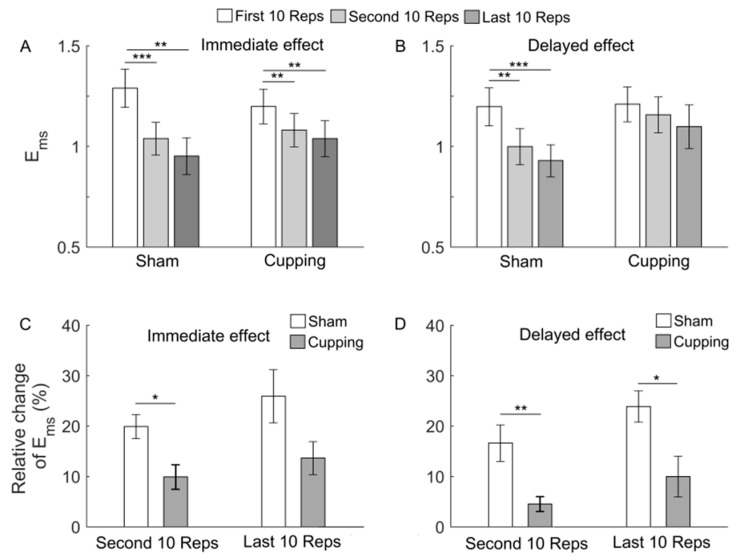
(**A**,**B**) $E_{ms}$ of the sEMG signals during the first, second and last 10 Reps of biceps curls. When exercise was performed immediately after sham control, the ANOVA yielded F = 4.77 and *p* = 0.0152; when exercise was performed immediately after cupping therapy, the ANOVA yielded F = 3.66 and *p* = 0.0367; when exercise was performed 24 h after sham control, the ANOVA yielded F = 5.72 and *p* = 0.0073; when exercise was performed 24 h after cupping, the ANOVA yielded F = 0.55 and *p* = 0.5803. (**C**,**D**) Relative changes of $E_{ms}$ during the second and last 10 Reps. The results are represented as mean ± standard error. ***, *p* < 0.001; **, *p* < 0.01; *, *p* < 0.05.

Figure 5A,B show $E_{ms}$ of the sEMG signals during the first, second, and last 10 Reps. When exercise was performed either immediately or 24 h after sham control, $E_{ms}$ showed a significant decrease from the first to second 10 Reps (*p* < 0.01). When exercise was performed after cupping therapy, the immediate effect was distinctly different from the delayed effect. In the later case, the decrease in $E_{ms}$ from the first to second 10 Reps was smaller than that in the former case.

Figure 5C,D compare the relative change in $E_{ms}$ during the first and last 10 Reps between sham control and cupping therapy. In the case of the immediate effect, during the second 10 Reps but not last 10 Reps, the relative change in $E_{ms}$ after cupping was significantly smaller than that after sham control (*p* < 0.05) (Figure 5C); in the case of delayed effect, during both second and last 10 Reps, the relative change in $E_{ms}$ after cupping was significantly smaller than that after sham control (*p* < 0.05) (Figure 5D).

**Figure 6:**
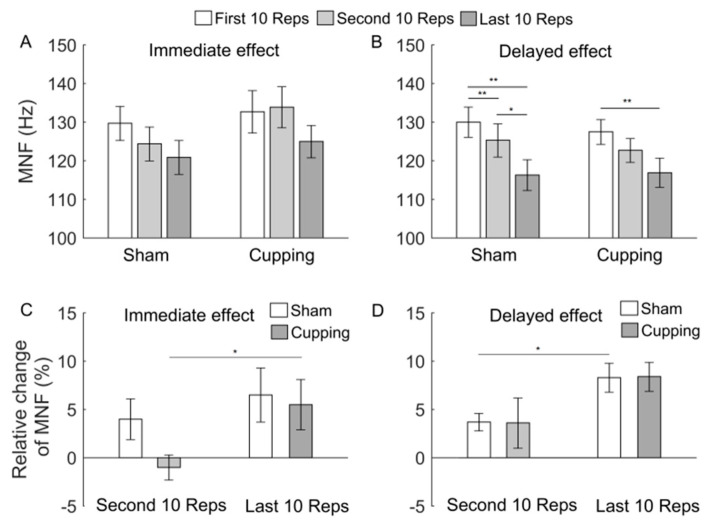
(**A**,**B**) MNF of the sEMG signals during the first, second and last 10 Reps of biceps curls. When exercise was performed immediately after sham control, the ANOVA yielded F = 0.99 and *p* = 0.3909; when exercise was performed immediately after cupping, the ANOVA yielded F = 0.92 and *p* = 0.4169; when exercise was performed 24 h after sham control, the ANOVA yielded F = 3.77 and *p* = 0.0361; when exercise was performed 24 h after cupping, the ANOVA yielded F = 4.4 and *p* = 0.0235. (**C**,**D**) Relative changes of MNF during the second and last 10 Reps. The results are represented as mean ± standard error. **, *p* < 0.01; *, *p* < 0.05.

**Figure 7:**
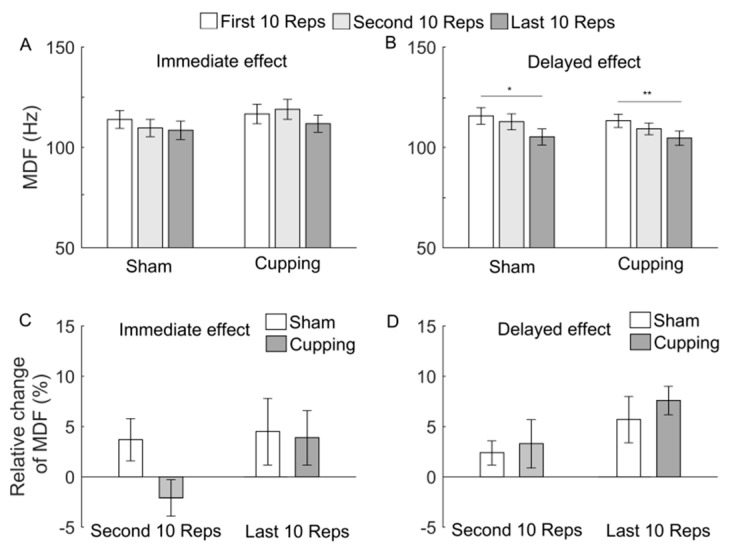
(**A**,**B**) MDF of the sEMG signals during the first, second and last 10 Reps of biceps curls. When exercise was performed immediately after sham control, ANOVA yielded F = 0.42 and *p* = 0.6628; when exercise was performed immediately after cupping, ANOVA yielded F = 0.62 and *p* = 0.5493; when exercise was performed 24 h after sham control, ANOVA yielded F = 3.46 and *p* = 0.0431; when exercise was performed 24 h after cupping, ANOVA yielded F = 3.87 and *p* = 0.0332. (**C**,**D**) Relative changes of MDF during the second and last 10 Reps. The results are represented as mean ± standard error. **, *p* < 0.01; *, *p* < 0.05.

**Figure 8:**
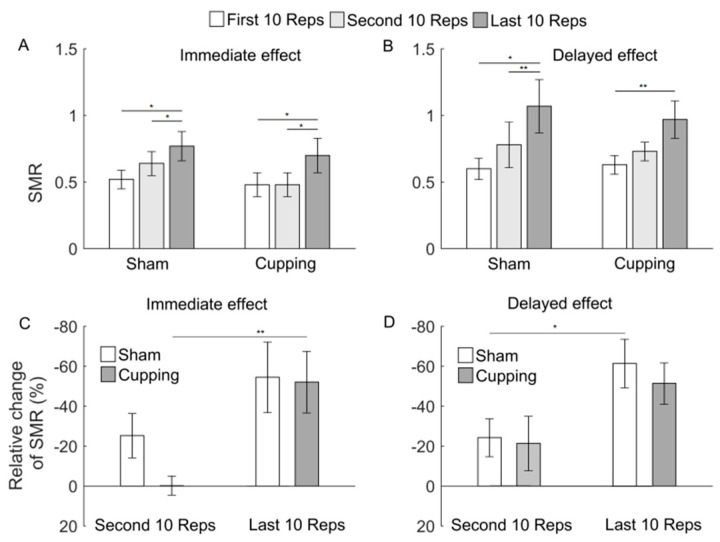
(**A**,**B**) SMR of the sEMG signals during the first, second and last 10 Reps of biceps curls. When exercise was performed immediately after sham control, the ANOVA yielded F = 3.95 and *p* = 0.0313; when exercise was performed immediately after cupping, the ANOVA yielded F = 3.42 and *p* = 0.0473; when exercise was performed 24 h after sham control, the ANOVA yielded F = 3.36 and *p* = 0.0499; when exercise was performed 24 h after cupping, the ANOVA yielded F = 4.34 and *p* = 0.0264. (**C**,**D**) Relative changes of SMR during the second and last 10 Reps. The results are represented as mean ± standard error. **, *p* < 0.01; *, *p* < 0.05.

Figure 6, Figure 7 and Figure 8 show the results of MNF, MDF, and SMR of the sEMG signals during the first, second, and last 10 Reps and their relative changes. When exercise was performed immediately after either sham control or cupping therapy, SMR showed a significant increase from the first to last 10 Reps (*p* < 0.05) and from the second to last 10 Reps (Figure 8A), whereas MNF and MDF did not show any significant change (Figure 6A and Figure 7A). When exercise was performed 24 h after sham control or cupping therapy, the three indices showed similar changes between two interventions (Figure 6B, Figure 7B and Figure 8B). The relative changes of the three indices did not show a significant difference between sham control and cupping therapy.

## 4. Discussion

This study indicates that the degree of regularity of sEMG signals quantified by $E_{ms}$ is capable of assessing muscle fatigue during dynamic contractions, as well as muscle recovery after cupping therapy. Moreover, $E_{ms}$ seems to be more sensitive to myoelectric alterations of muscle fatigue compared to the traditional linear indices.

In this study, the methodological selection was motivated by the following considerations. The underlying mechanisms of sEMG generation have been found to be nonlinear or even chaotic in nature [33], manifesting as complex patterns of the sEMG signal, which can be influenced by many factors such as muscle fatigue [9]. There is evidence that muscle fatigue usually leads to a loss of complexity of the sEMG signal [34]. This means that complexity analysis is a promising tool in assessing muscle fatigue during a motor task. However, complexity analysis methods generally require very large datasets to yield reliable estimations. In this study, since the amplitudes of sEMG signals during the intervals between two successive repetitions were almost zero (Figure 3B), it may be unsuitable to directly apply any linear or nonlinear measures to the sEMG signal across 10 Reps because of its high non-stationarity. To address this problem, we considered the signal segments during each repetition (Figure 4). Since the duration of a repetition was approximately 4 s, given the exercise tempo of 15 Reps per minute, the length of a signal segment was approximately 4000. Such a length requires the selected measure to be robust to short time series. As demonstrated earlier and in our previous studies [19], $E_{ms}$ is insensitive to record length, independent of sampling rates, and relatively consistent for varying parameters. Therefore, we employed $E_{ms}$ to quantify the degree of regularity of sEMG signals.

Our results showed that when biceps curls were performed either immediately or 24 h after sham control, $E_{ms}$ of the sEMG signal underwent a significant decrease from the first to second set of exercise (Figure 5). This means that a significant enhancement of regularity of the sEMG signal occurred during the second 10 Reps. According to the literature, a feature of central fatigue is a shift of the recruitment of motor units toward a more synchronized pattern [9]. Hence, our results suggested that the significant decrease in $E_{ms}$ of the sEMG signal was a myoelectric manifestation of muscle fatigue.

It should be noticed that after sham control, changes in $E_{ms}$ (Figure 5) were roughly consistent with but different from changes in MNF, MDF, and SMR (Figure 6, Figure 7 and Figure 8). For instance, from the first to second 10 Reps, $E_{ms}$ showed a significant decrease in both conditions, i.e., exercise performed immediately and 24 h after sham control (Figure 5), whereas MDF and SMR showed only slight changes in both conditions (Figure 7 and Figure 8), and MNF showed a significant change in the later condition but not in the former condition (Figure 6). This observation suggested that $E_{ms}$ was more sensitive to myoelectric alterations of muscle fatigue and yielded more consistent results compared to MNF, MDF, and SMR. One possible reason for such a discrepancy was that $E_{ms}$ and the spectral indices reflect different aspects of the electromyographic properties. On one hand, fatigue causes a shift of motor unit recruitment toward a more synchronized pattern, which, in turn, results in more regular structures of the sEMG signal [35,36]. On the other hand, fatigue causes a decrease in conduction velocity of action potentials, which leads to a relative shift in the energy of muscle contractions from high to low frequencies [9].

Our results also showed that during the last 10 Reps, the immediate effect of cupping therapy on relative change in $E_{ms}$ did not significantly differ from that of sham control (Figure 5C), whereas the delayed effect of cupping therapy was manifested as a significant smaller relative change in $E_{ms}$ compared to sham control (Figure 5D). If one assumes that Sample Entropy is more sensitive to myoelectric alterations of muscle fatigue than MNF, MDF, and SMR also after cupping therapy, our results suggest that the delayed effect of cupping on reducing muscle fatigue was superior to the immediate effect. One possible reason may be that muscle fatigue caused by biceps curls could not be completely removed immediately after cupping therapy. It is well known that exercise produces intramuscular H^+^ and leads to an accumulation of H^+^ within the muscle, which can eventually cause muscle fatigue [9]. Cupping therapy can increase tissue blood-flow by eliciting vasodilation and petechiae [37] and, thus, may increase clearance of H^+^. However, the recovery of elevated muscle pH caused by intensive exercise to the pre-exercise level may take one hour or longer time [38]. In this study, cupping therapy lasted for 5 min. Such a duration may not be adequate to remove H^+^ completely. On the other hand, the superiority of the delayed effect of cupping therapy may be associated with a delayed inflammatory response. In this study, biceps curls involved eccentric muscle contractions, which can exert large mechanical stress on the myofibrils and induce muscle damage and, therefore, trigger inflammation [39]. Additionally, cupping therapy can lead to capillary rupture and ecchymosis and induce inflammatory response [40]. Hence, cupping therapy could aggravate the inflammation caused by intensive exercise. This may partially explain the superiority of delayed effect of cupping therapy.

**Figure 9:**
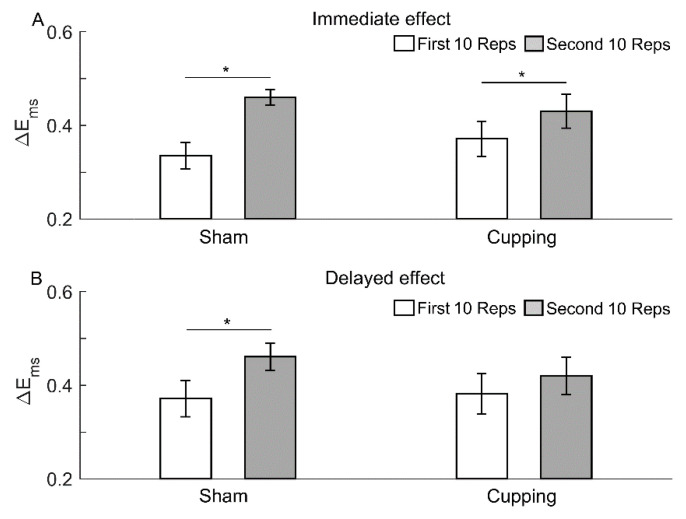
Difference in $E_{ms}$ between the sEMG signal during the first and second 10 Reps and surrogate data. The results are represented as mean ± standard error. *, *p* < 0.05 (paired *t*-test). For each segment of the sEMG signal during the first and second 10 Reps, $E_{ms}$ was computed for 30 phase-randomized surrogate time series. Then, the $E_{ms}$ values were averaged across 30 surrogate time series and across 10 segments. (**A**) Immediate effect. (**B**) Delayed effect.

To get an insight into the role of nonlinearity of sEMG during the development of muscle fatigue and into the influence of cupping therapy on nonlinearity of sEMG, we performed the following experiments. For each segment of the sEMG signal during the first and second 10 Reps, we computed $E_{ms}$ for 30 phase-randomized surrogate time series. Then, the $E_{ms}$ values were averaged across 30 surrogate time series and across 10 segments. The difference in $E_{ms}$ between the sEMG signal and surrogate time series, denoted as $\Delta E_{ms}$, reflects the nonlinearity of the signal. Figure 9 shown the statistical results of $\Delta E_{ms}$ during the first and second 10 Reps in all subjects. By comparing Figure 9 and Figure 5, it could be deduced that immediately or 24 h after sham control or immediately after cupping therapy, the significant decrease in $E_{ms}$ of the sEMG signal from the first to second 10 Reps was largely attributed to a loss of nonlinearity of the signal.

This study had several limitations. First, the load of biceps curls was selected as 75% of 10 RM based on the consideration of exercise safety and the assumption that such a load could cause significant changes in sEMG features. Future studies may need to examine changes in sEMG features under other exercise loads such as 60–75% of 10 RM, 1 RM, and 5 RM. Second, cupping therapy was conducted immediately after intensive exercise. Future studies may need to identify the optimal time-point of cupping therapy for reducing exercise-induced muscle fatigue. Last, it is unclear whether cupping therapy may change the skin condition for affecting sEMG signals. In our previous studies, cupping therapy has been demonstrated to reduce muscle stiffness assessed by elastographic ultrasound [20] and improve skin blood-flow assessed by laser Doppler flowmetry [21,23]. Under these conditions, the amplitude of sEMG signals may be changed. However, spectral analysis and entropy based analysis of muscular fatigue may not be significantly affected by the alterations of the EMG amplitudes. The change of median frequency and complexity of sEMG signal reflect the spectral component and nonlinear complexity that may not be significantly correlated to the skin condition altered after cupping therapy. Nevertheless, future studies may examine the effect of skin condition on the complexity of sEMG signals.

## 5. Conclusions

The present study indicated that the degree of regularity of sEMG signals quantified by $E_{ms}$ is capable of detecting myoelectric alterations of muscle fatigue and, therefore, can be used to assess muscle fatigue during dynamic contractions and the effect of cupping therapy. Moreover, this measure showed a higher sensitivity to muscle fatigue and yielded more consistent results compared to the traditional linear measures such as MNF, MDF, and SMR.

## Data Availability

The data are available from the corresponding author upon a reasonable request.
